# Mechanisms underlying synergism between circularized tumor necrosis factor‐related apoptosis inducing ligand and bortezomib in bortezomib‐sensitive or ‐resistant myeloma cells

**DOI:** 10.1002/hon.3045

**Published:** 2022-07-14

**Authors:** Yun Leng, Xiaoyan Hu, Lin Li, Jewel Nkwocha, Toshihisa Satta, Kanika Sharma, Maciej Kmeiciak, Huixing Zhou, Zhiyao Zhang, Liang Zhou, Wenming Chen, Steven Grant

**Affiliations:** ^1^ Department of Hematology Beijing Chao‐Yang Hospital Capital Medical University Beijing China; ^2^ Division of Hematology/Oncology Department of Medicine Virginia Commonwealth University Richmond Virginia USA; ^3^ Massey Cancer Center Virginia Commonwealth University Richmond Virginia USA

**Keywords:** bortezomib, CPT, intrinsic/extrinsic apoptotic, multiple myeloma, non‐canonical NF‐κB pathway, TRAIL

## Abstract

Mechanisms underlying interactions between a novel, clinically relevant circularized tumor necrosis factor‐related apoptosis inducing ligand (TRAIL) agonist, circularly permuted TRAIL (CPT) have been examined in multiple myeloma (MM) cells sensitive or resistant to bortezomib (BTZ). Various MM cell lines for example, U266, including those resistant to bortezomib‐resistant U266 cells were exposed to low nanomolar concentrations of bortezomib ± CPT and apoptosis monitored. Circularly permuted TRAIL and bortezomib synergistically induced apoptosis in both BTZ‐naïve and ‐resistant cells. The regimen up‐regulated DR4 receptor internalization in MM cells, known to modulate both NF‐κB and extrinsic apoptotic pathways. CPT/BTZ disrupted the non‐canonical NF‐κB pathway, reflected by tumor necrosis factor (TNF) receptor associated factors 3 (TRAF3) up‐regulation, NF‐κB inducing kinase down‐regulation, diminished p52 and p50 processing, and B‐cell lymphoma‐extra large (BCL‐XL) down‐regulation, but failed to inactivate the canonical NF‐κB pathway, reflected by unchanged or increased expression of phospho‐p65. The regimen also sharply increased extrinsic apoptotic pathway activation. Cells exhibiting TRAF3 knock‐down, dominant‐negative Fas‐associated protein with death domain, knock‐down of caspase‐8, BCL‐2/BCL‐XL, or exposure to a caspase‐9 inhibitor displayed markedly reduced CPT/BTZ sensitivity. Concordant results were observed in bortezomib‐resistant cells. The regimen was also active in the presence of stromal cells and was relatively sparing toward normal CD34^+^ hematopoietic cells. Finally, ex vivo results revealed synergism in primary MM primary cells, including those BTZ, and the CPT/BTZ regimen significantly decreased tumor growth in a patient‐derived MM xenograft model. These results indicate that the CPT/BTZ regimen acts via the non‐canonical NF‐κB as well as intrinsic/extrinsic apoptotic pathways to induce cell death in MM cells, and may represent an effective strategy in the setting of bortezomib resistance.

## 1 INTRODUCTION

Multiple myeloma (MM) is a clonal disorder of plasma cells^1^ accounting for 13% of hematologic malignancies.^2^ Bortezomib (BTZ) is a proteasome inhibitor which promotes MM apoptosis,^3^, ^4^ possibly by inhibiting the NF‐κB pathway, upon which MM cells depend.^5^ The complete remission rate for bortezomib‐based regimens is as high as 30%, and the progression‐free survival of MM patients is significantly prolonged.^6^ However, relapse and bortezomib resistance represent significant problems in MM.^7^ Notably, BTZ‐resistant cells have shown increased NF‐κB signaling.^8^

Tumor necrosis factor‐related apoptosis inducing ligand (TRAIL) induces cell death in diverse malignant cells, but spares normal cells.^9^ It has shown pre‐clinical activity in MM, through various mechanisms, including disruption of the canonical and non‐canonical NF‐κB pathways, and activation of the extrinsic apoptotic cascade.^9^ It also interacts synergistically with proteasome inhibitors.^10^, ^11^ TRAIL has limited activity in the clinic, possibly through failure of target engagement.^12^ This led to the development of circularly permutated TRAIL (CPT), a cyclization allosteric form of wild‐type TRAI.^13^ Compared to wild‐type TRAIL, CPT has greater antitumor activity, and better stability/biological activity in aqueous solution.^14^ In a phase II trial of CPT monotherapy, among 27 MM patients with relapsed/refractory multiple myeloma (RRMM), 1 patient achieved a near‐complete response, and 8 patients achieved partial responses.^15^ Another phase II clinical trial of CPT combined with thalidomide and dexamethasone showed promise.^16^ Here, our goals were to determine whether the CTP/BTZ regimen was effective in BTZ‐sensitive or ‐resistant MM, including in a MM patient‐derived MM xenograft (PDX) model system, and to elucidate mechanisms underlying interactions.

## 2 MATERIALS AND METHODS

### 2.1 Cell lines and reagents

Multiple myeloma cell lines U266, bortezomib‐resistant U266 cells (PS‐R), Roswell Park Memorial Institute (RPMI) 8226, H929, U266/Fas‐associated protein with death domain dominant negative (FADD‐DN) cell line, U266/C8‐DN cell line, U266/pcDNA3.1 cell line, U266/shRNA targeting TNF receptor associated factor 3 (shTRAF3) determinant domain silenced cells, U266/BCL‐2, U266/BCL‐X_L_ and HS‐5 cells were used as previously described.^17^

Circularly permuted TRAIL was obtained from Beijing Shandong Biotechnology Co., Ltd. and a solution was prepared by diluting CPT100 mg to 1 mg/ml with 0.1% bovine serum albumin in phosphate buffered saline (PBS), which was stored at −80°C. Bortezomib (Spectrum Pharmaceuticals) solution was prepared by diluting bortezomib (PS‐341; molecular weight = 384.24) with dimethyl sulfoxide to 10 mM, and stock solutions diluted in RPMI to achieve the desired final concentration.

## 3 ISOLATION OF PRIMARY MYELOMA CELLS

All studies were obtained with written informed consent from patients undergoing routine diagnostic aspirations, were conducted in accordance with recognized ethical guidelines (e.g., the Declaration of Helsinki), and were approved by the Virginia Commonwealth Institutional Review Board (#MCC‐8712‐3A; MCC‐02447; MCC‐03340) and the Ethics Committee of Beijing Chaoyang Hospital, Capital Medical University (Reference: 2014‐y‐76). Medical records were de‐identified, and only information relating to pre‐biopsy treatment was reviewed.

CD138^+^ bone marrow cells from 13 patients with RRMM were purified by CD138 microbeads using a Miltenyi magnetic cell sorting system. The purity of the myeloma cells assessed by CD138/CD45 staining and morphology was ≥95%.

### 3.1 Animal studies

Animal studies were conducted under an approved protocol by the local Ethics Committee of both participating institutions and complied with the institutional guidelines for the care and use of animals. The establishment of a PDX model is described in Supplemental data. Relapsed/refractory multiple myeloma patient‐derived mononuclear cells were injected subcutaneously into the upper limbs of SPF‐grade nonobese diabetic‐severe combined immunodeficiency female mice (3–4 weeks of age) with 10 × 10^6^ cells/100 μl. When the tumor size was approximately 40–110 mm^3^, 20 tumor‐bearing mice were randomly divided into four groups (5 mice/group), for example, control group, BTZ single‐drug group, CPT single‐drug group, and the combination group. Mice were injected subcutaneously with BTZ 0.5 mg/kg (at day 1, 4, 8 and 11) ± CPT 10 mg/kg (daily). Control mice were subcutaneously injected with PBS. Tumor size and the general mouse conditions were measured twice/week. After 5 weeks, mice were sacrificed and tumor volumes calculated by the formula *V* = (width *x* length^2^)/2.

### 3.2 Statistical analysis

Values represent the means ± standard deviation for at least three independent experiments performed in triplicate. The significance of differences between experimental variables was determined by using the Student *t* test or one‐way analysis of variance with the Tukey‐Kramer multiple comparisons test. The significance of *p* values is indicated: *p < 0.05, **p < 0.01, or ***p < 0.001. The combination index (CI) value was calculated according to Compusyn 1.0 software. CI < 1, = 1 and > 1 indicated that the two drugs had synergistic, additive and antagonistic effects, respectively.^18^

## 4 RESULTS

### 4.1 CPT interacts synergistically with BTZ in MM cells

**FIGURE 1:**
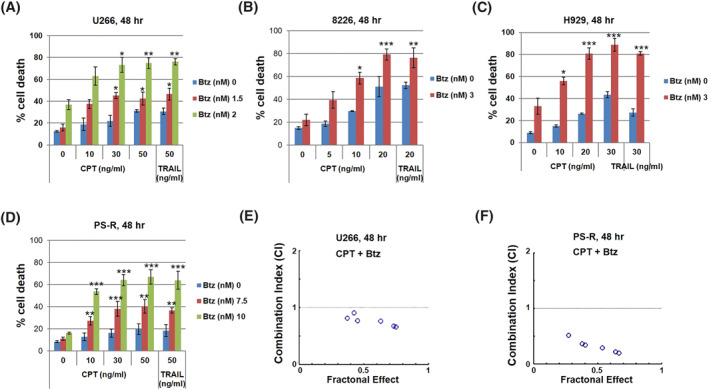
The circularly permutated TRAIL (CPT)/bortezomib regimen synergistically induces apoptosis in multiple myeloma (MM) cells. (A‐D) U266, 8226, H929 and bortezomib‐resistant U266 cells (PS‐R) cells were exposed (48 h) to indicated doses of CPT and resistant to bortezomib (BTZ) treatment, followed by flow cytometric analysis of cell death after staining with 7‐AAD. The percentages of 7‐AAD (+) cells are presented. (E and F) U266 and PS‐R cells were exposed (24 h) to varying concentrations of CPT ± BTZ at a non‐fixed ratio, after which the percentage of 7‐AAD^+^ cells was determined. Combination Index (CI) values less than 1.0 denote a synergistic interaction; *p < 0.05; **p < 0.01; ***p < 0.001

Minimally toxic concentrations of BTZ (1.5–3 nM) with CPT (20–50 ng/ml) or TRAIL (20–50 ng/ml) significantly increased lethality, reflected by 7‐AAD uptake, in various MM cells, for example, U266, 8226, and H929 (Figure 1A–C) and highly BTZ‐resistant PS‐R cells,^19^ although requiring higher BTZ concentrations (e.g., 7.5–10 nM) (Figure 1 D). Median Dose Effect analysis yielded CI values substantially less than 1.0, indicating synergism (Figure 1E,F lower panels).

### 4.2 Combined treatment with CPT and BTZ promotes pronounced DR4 receptor internalization

To determine whether CPT/BTZ activity specifically involved DR4/5, U266 and PS‐R cells were incubated with CPT (30 ng/ml) and BTZ (2 nM) for16 h, and stained with DR4 or DR5 antibodies. Immunofluorescence microscopy showed strong internalization of the DR4 receptor in both U266 and PS‐R cells exposed to both agents (Supplemental Figure S1A). In contrast, this was not seen for DR5 (Supplemental Figure S1B), suggesting that the CPT/BTZ regimen activates DR4‐ (but not DR5‐) induced apoptosis.

### 4.3 The CPT/BTZ regimen activates the extrinsic apoptotic pathway

**FIGURE 2:**
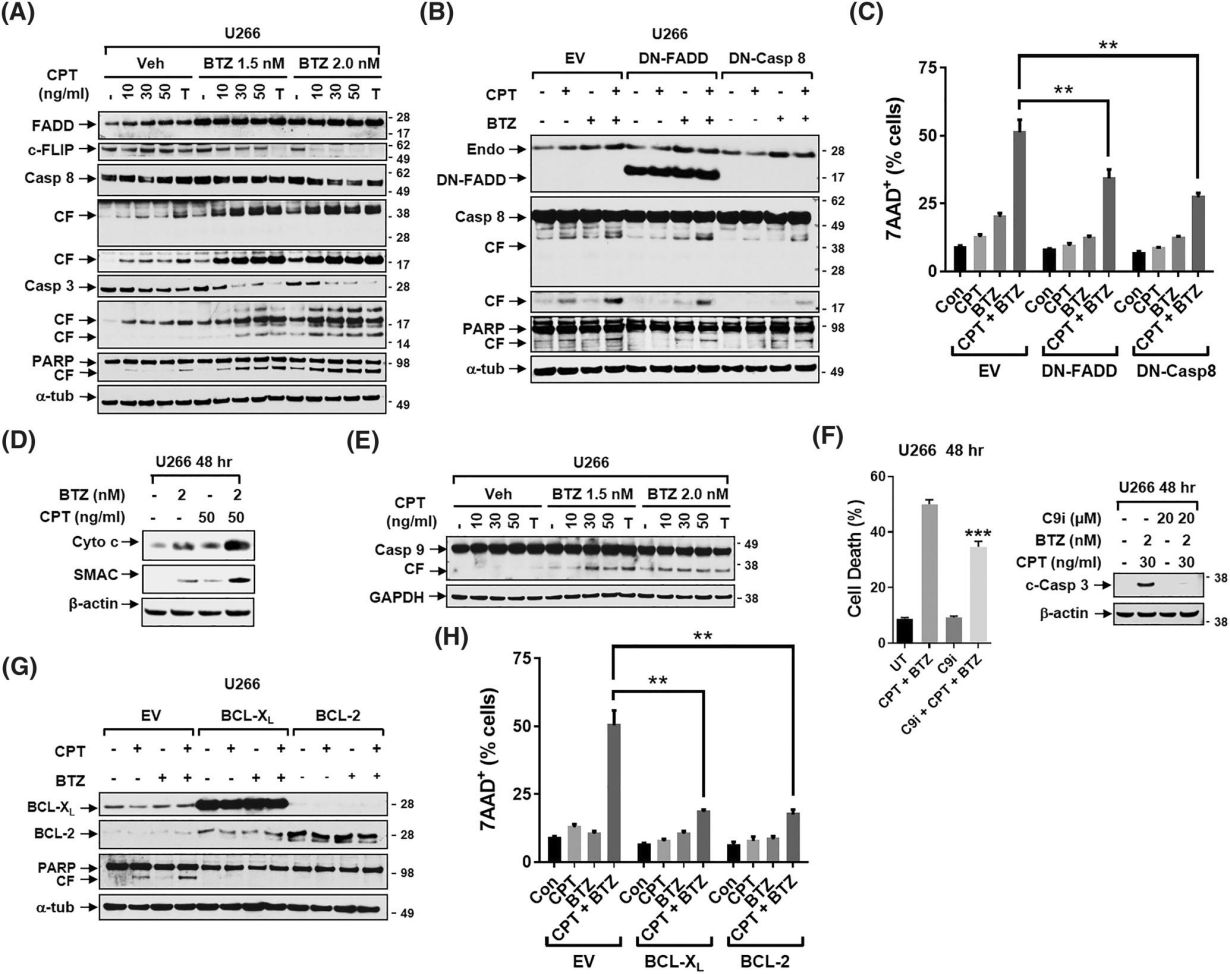
The circularly permutated TRAIL (CPT)/resistant to bortezomib (BTZ) regimen activates of the extrinsic/intrinsic apoptotic pathway. (A) U266 were incubated with CPT ± BTZ for 48 h. Caspase‐8, Caspase‐3, poly‐ADP ribose polymerase (PARP), Fas‐associated protein with death domain (FADD), and cellular FLICE‐inhibitory protein (c‐FLIP) were monitored by immunoblotting analysis. CF = cleavage fragment. α‐tubulin was assayed to ensure equivalent loading and transfer. (B) U266/EV, U266/DN‐FADD and U266/DN‐Casp 8 were treated with the indicated concentrations of CPT ± BTZ for 48 h. FADD, Caspase 8 and PARP were monitored by immunoblotting analysis. CF = cleavage fragment. α‐tubulin was assayed to ensure equivalent loading and transfer. (C) Cells were treated as mentioned in B, followed by flow cytometric analysis of cell death after staining with 7‐AAD. The percentages of 7‐AAD (+) cells are presented. (D) U266 cells were incubated with indicated doses of CPT ± BTZ for 48 h. Cytochrome C and second mitochondrial activator of caspases (SMAC) were monitored by immunoblotting analysis. (E) U266 cells were incubated with of CPT ± BTZ for 48 h. Caspase 9 monitored by immunoblotting analysis. (F, left panel) U266 cells were pre‐treated for 30 min with caspase nine inhibitor (Z‐LEHD‐FMK, 20 μM) and then incubated with BTZ (2 nM) + CPT (30 ng/ml) for 48 h. After treatment, cells were subjected to flow cytometry to determine the percentage of death (7‐AAD^+^ cells). (F, right panel) Immunoblotting analysis was then performed to monitor levels of cleaved Caspase‐3. (G) U226/EV, U226/BCL‐X_L_, and U226/BCL‐2 cells were incubated with CPT (10 ng/ml) ± BTZ (3 nM) for 48 h. BCL‐X_L_, BCL‐2 and PARP were monitored by immunoblotting analysis. CF = cleavage fragment. (H) The percentages of 7‐AAD (+) cells are presented. Glyceraldehyde‐3‐Phosphate Dehydrogenase, β‐actin, or α‐tubulin was assayed to ensure equivalent loading and transfer. **p < 0.01; ***p < 0.001

**FIGURE 3:**
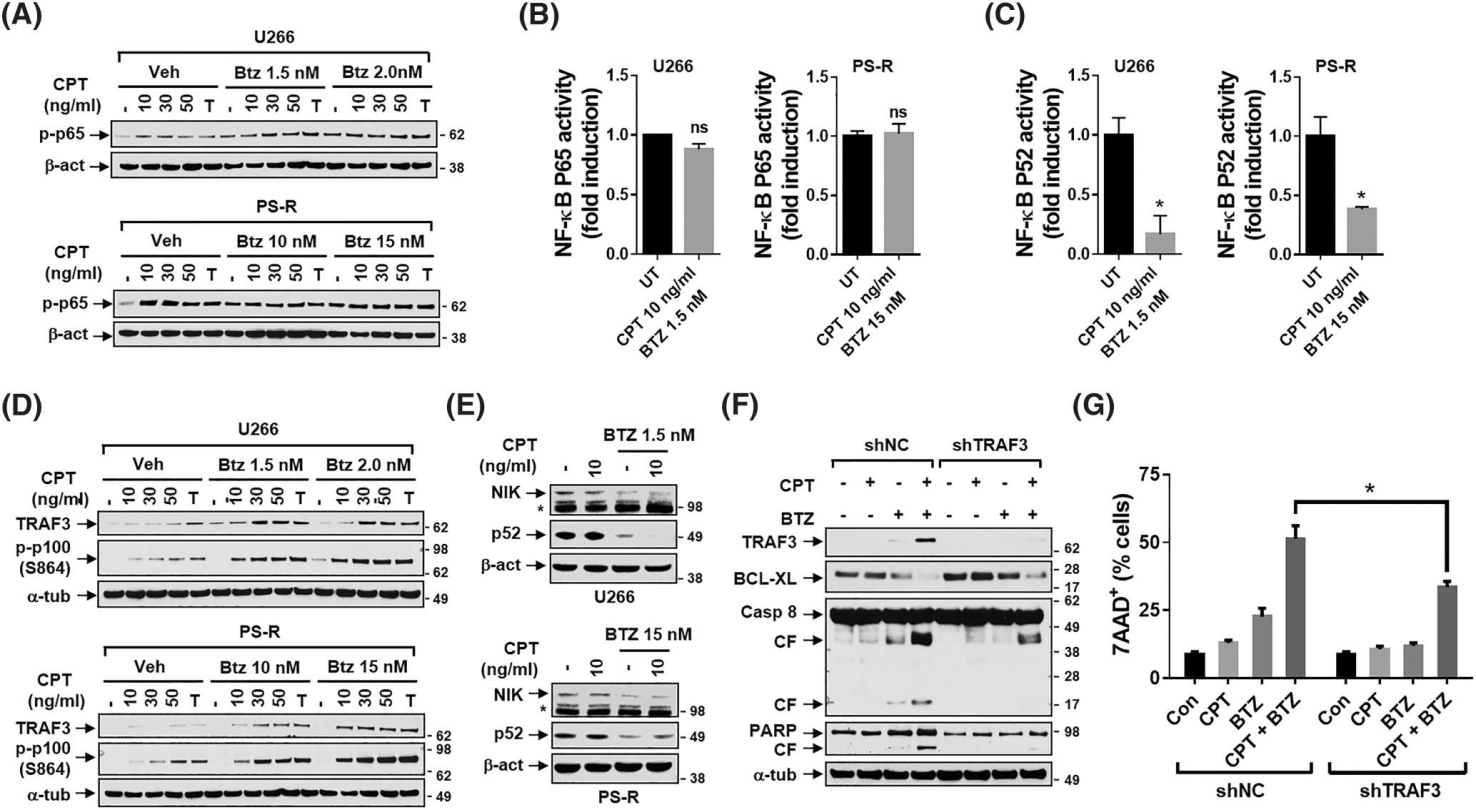
The circularly permutated TRAIL (CPT)/resistant to bortezomib (BTZ) regimen inhibits non‐canonical NF‐κB signaling pathway (A,B) U266 or PS‐R cells were incubated with CPT ± BTZ for 48 h (A) p‐p65 (S536) was monitored by immunoblotting analysis. (B) DNA binding of NF‐kB (p65 subunit) was determined by using a TransAM assay for NF‐kB. (C) U266 or PS‐R cells were treated with CPT ± BTZ for 40 h. DNA binding of NF‐kB (p52 subunit). (D) Cells were treated as mentioned in A. TNF receptor associated factors 3 (TRAF3) and p‐p100/p52 (S864) were monitored by immunoblotting analysis. (E) U266 or PS‐R cells were treated with indicated doses of CPT ± BTZ for 48 h. NF‐κB inducing kinase (NIK) and p52 were monitored by immunoblotting analysis. (F) U266/shNC and U266/shRNA targeting TNF receptor associated factor 3 (shTRAF3) cells were exposed to the indicated concentrations of CPT ± BTZ for 48 h. Immunoblotting analysis was then performed to monitor levels of TRAF3, B‐cell lymphoma‐extra large (BCL‐X_L_), Caspase‐8 and poly‐ADP ribose polymerase (PARP). CF = cleavage fragment. α‐tubulin or β‐actin was assayed to ensure equivalent loading and transfer. (G) Cells were treated as mentioned in F, followed by flow cytometric analysis of cell death after staining with 7‐AAD. The percentages of 7‐AAD (+) cells are presented. *p < 0.05

Exposure (24 h) of U266 cells to low, minimally toxic concentrations of CPT (10–50 nM) or TRAIL and very low BTZ concentrations (1.5 or 2 nM), western blot analysis demonstrated that combined treatment induced marked caspase 8/caspase‐3/poly‐ADP ribose polymerase (PARP) cleavage, Fas‐associated protein with death domain (FADD) up‐regulation, and down‐regulation of cellular FLICE‐inhibitory protein (c‐FLIP), a master anti‐apoptotic regulator (Figure 2A). Similar results were observed in highly BTZ‐resistant PS‐R cells exhibiting high basal c‐FLIP levels, using modestly higher BTZ concentrations (e.g., 10–15 nM) (Supplemental Figure S2A). To assess the functional role of the extrinsic apoptotic pathway in CPT/BTZ responses, U266 cells ectopically expressing dominant‐negative FADD or caspase‐8 (DN‐FADD or DN‐Casp 8) showed dramatically reduced caspase eight and PARP cleavage compared to controls (Figure 2B). Both DN‐FADD and DN‐Casp eight expression significantly diminished CPT/BTZ‐induced cell death (**p < 0.01; Figure 3D), arguing that the extrinsic apoptotic pathway contributes to CPT/BTZ activity in BTZ‐sensitive or ‐resistant MM cells.

### 4.4 The CPT/BTZ regimen activates the intrinsic apoptotic pathway

To investigate the role of the intrinsic apoptosis in CPT/BTZ anti‐MM activity, U266 and PS‐R cells were treated with indicated concentrations of CPT or TRAIL ± BTZ for 48 h. Combined exposure to both agents sharply increased cytochrome C and second mitochondrial activator of caspases release, accompanied by caspase‐9 cleavage (Figure 2D,E; Supplemental Figure S2B, 2C). The caspase‐9 inhibitor Z‐LEHD‐FMK(C9i) significantly blocked CPT/BTZ–induced caspase activation and cell death (Figure 2F). To investigate the effect of anti‐apoptotic family members (e.g., BCL‐2 and BCL‐X_L_), U266 cells ectopically expressing BCL‐X_L_ or BCL‐2 and exposed to CPT/BTZ displayed reduced PARP cleavage (Figure 2G) and significantly diminished cell death compared to controls (Figure 2H), arguing that intrinsic apoptosis contributes functionally to CPT/BTZ activity in MM cells.

### 4.5 The CPT/BTZ regimen inhibits non‐canonical but not canonical NF‐κB signaling

As MM cell survival is partly dependent upon NF‐κB activation,^20^ CPT/BTZ effects on NF‐κB pathways were examined in U266 and PS‐R cells. Exposure to CPT ± BTZ, failed to down‐regulate (or increase) p65 phosphorylation (S536), reflecting canonical NF‐κB activation,^21^ in both cell types (Figure 3A). An NF‐κB p65 DNA binding assay confirmed that CPT/BTZ failed to increase p65 binding activity in U266 or PS‐R cells (Figure 3B), arguing that this regimen acts independently of the canonical NF‐κB pathway.

In sharp contrast, CPT/BTZ significantly reduced NF‐κB p52 binding activity determined by a p52 Chemi Act Assay (Figure 3C), an indicator of non‐canonical NF‐κB activation.^22^ In both U266 and PS‐R cells, CPT/BTZ clearly increased TNF receptor associated factors 3 (TRAF3) expression, a negative regulator of the non‐canonical pathway,^22^ accompanied by increased p100 expression (Figure 3D; S864), presumably reflecting diminished cleavage to the active p52 form. In both U266 and PS‐R cells, CPT/BTZ induced down‐regulation of NF‐κB inducing kinase (NIK) (NF‐κB‐initiating kinase), a non‐canonical NF‐κB pathway activator^23^ and downstream inhibitory target of TRAF3^24^ (Figure 3E).

To evaluate TRAF3 up‐regulation functionality, U266 cells ectopically expressing TRAF3 knockdown (shTRAF3) were generated. Following CPT/BTZ treatment, shTRAF3 cells exhibited markedly diminished Caspase 8 and PARP cleavage. TNF receptor associated factors 3 knockdown cells exhibited up‐regulation of BCL‐X_L,_ a key down‐stream target of the non‐canonical pathway,^25^ and diminished down‐regulation following CPT/BTZ treatment (Figure 3F). They also displayed reduced caspase 8 cleavage/activation (Figure 3F). Finally, TRAF3 knockdown cells were significantly less susceptible to CPT/BTZ than empty‐vector controls (*p < 0.05, Figure 3G). These findings indicate that disruption of the non‐canonical (but not the canonical) NF‐κB pathway plays a significant functional role in CPT/BTZ‐mediated anti‐myeloma activity.

### 4.6 CPT/BTZ circumvents microenvironment‐driven intrinsic resistance

Co‐culture of GFP‐labeled PS‐R cells with human stromal cells (HS‐5) failed to protect cells following 48 h exposure to CPT/BTZ (Fig. Supplemental Figure S3A). Fluorescence microscopy images revealed a marked increase in 7‐AAD staining (red) after treatment of GFP‐labeled PS‐R cells cultured with HS‐5 cells with the CPT/BTZ regimen (Supplemental Figure S3B), arguing that CPT/BTZ exposure is lethal to BTZ‐resistant MM cells cultured with human stromal cells.

### 4.7 The CPT/BTZ regimen is active against primary CD138^+^ MM cells

To examine CPT/BTZ activity against primary CD138^+^ MM cells, 13 patient specimens were investigated. Detailed clinical/pathologic features are shown in Supplemental Table S1. All but two patients were relapsed/refractory (RR), each of whom had received bortezomib. The median number of prior cycles of bortezomib was 6 (range 2–9), and the median number of cycles was 7 (range 1–24). 5/13 specimens were positive for TP53 deletion by fluorescence in situ hybridization (FISH), 3/13 patients had 1q21 amplification, and 2 had a complex karyotype (Supplemental Figure S4).

**FIGURE 4:**
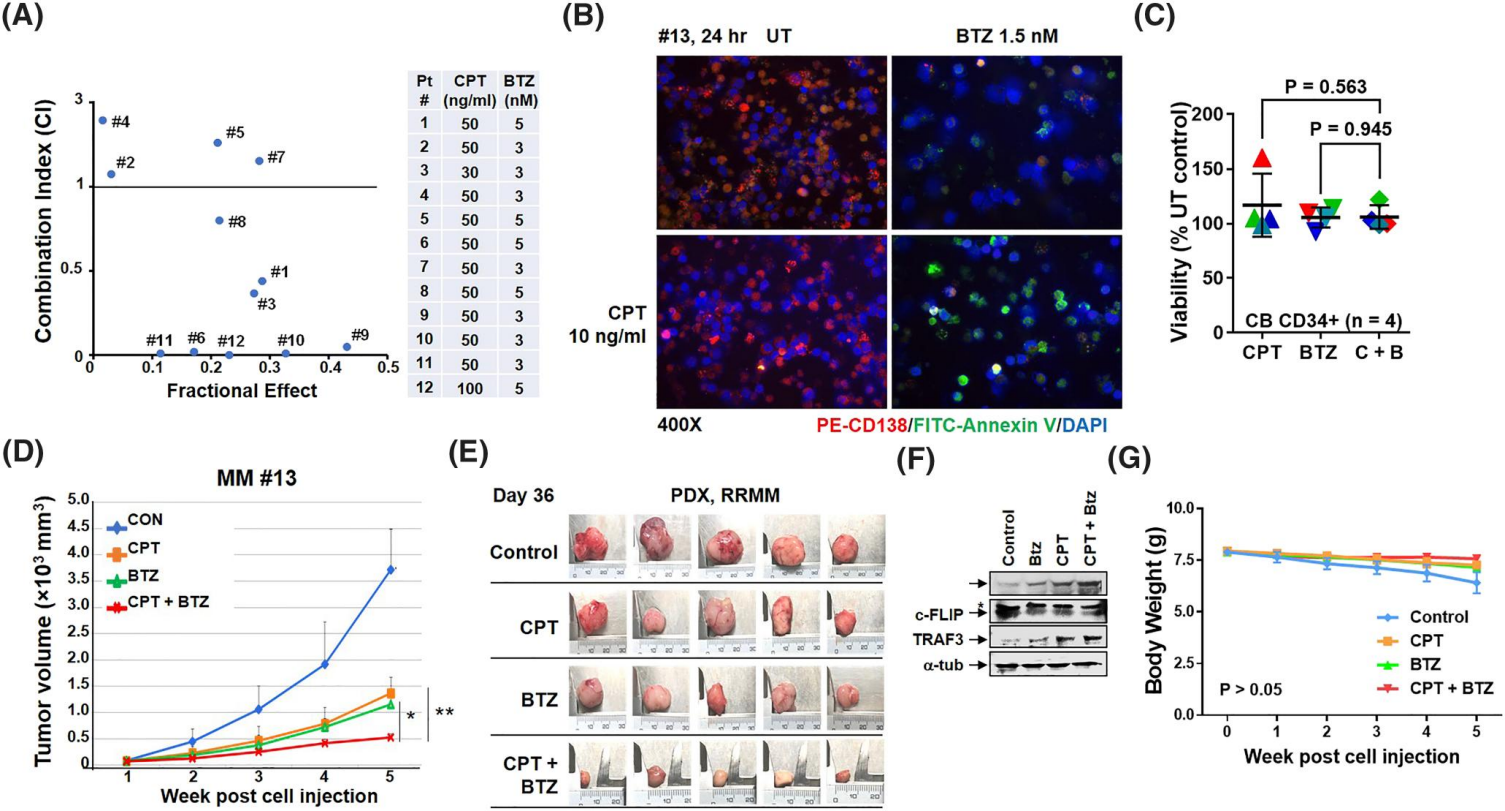
The circularly permutated TRAIL (CPT)/resistant to bortezomib (BTZ) regimen inhibits primary multiple myeloma (MM) cell growth ex vivo and MM cell growth in vivo. (A) Patient‐derived bone marrow mononuclear cells were isolated and treated with indicated doses of CPT ± BTZ for 24 h, after which the cells were stained with CD138‐PE. Flow cytometric analysis was performed to determine the CD138+ population. Combination index (CI) values less than 1.0 denote a synergistic interaction. (B) Representative primary bone marrow cells from a patient with MM (RR, relapse and refractory; prior BTZ) were exposed to 10 ng/ml CPT +/− 1.5 nM BTZ for 24 h, after which the cells were stained with CD138‐PE and annexin V‐fluorescein isothiocyanate (FITC). Images were obtained with an IX71‐Olympus inverted system microscope at × 40 magnification. (C) Experiments were carried out with 4 primary cord blood (CB) CD34^+^ samples. *p* > 0.05. (D–G) 20 SPF‐grade NOD‐SCID mice were inoculated via flank s.c. with 10 × 10^6^ patient‐derived MM cells. Mice were randomized to 4 groups (*n* = 5/group). Treatment was initiated after the tumor size was about 40–100 mm^3^. Mice were administered subcutaneously with BTZ 0.5 mg/kg (at day 1, 4, 8 and 11) ± CPT 10 mg/kg (daily). Tumor growth and body weight were monitored weekly (D, G). At day 36, tumors were harvested and dissected into small pieces. Immunoblotting analysis was then performed to monitor levels of c‐PARP, cellular FLICE‐inhibitory protein (c‐FLIP), and TNF receptor associated factors 3 (TRAF3). α‐tubulin was assayed to ensure equivalent loading and transfer (E, F)

Following 24 h exposure of primary MM cells to CPT and BTZ alone or in combination, cell death was determined by annexin/V staining and subjected to Median Dose Effect analysis. 8/12 specimens displayed CI values < 1.0, corresponding to synergistic interactions (Figure 4A). Fluorescence microscopy of cells from a representative specimen illustrates the pronounced increase in green (annexin V) staining of cells exposed to both agents (Figure 4B). Comparable exposure of normal CD34^+^ cord blood samples (*N* = 4) failed to reduce viable cell numbers after single or combined drug treatment (Figure 4C).

## 5 THE CPT/BTZ REGIMEN IS ACTIVE IN A PRIMARY, PATIENT‐DERIVED MM XENOGRAFT MODEL

To assess CPT/BTZ activity in vivo, a PDX model was established using cells obtained from a patient with RRMM (Supplemental Table S1). Primary ascites tumor cell suspensions containing 10 × 10^6^ cells/100 μl were injected subcutaneously into mouse upper limbs. After 4 weeks, transplanted tumors were resected, manipulated into a single‐cell suspensions, and analyzed by FISH, the results of which are shown in Supplemental Figure S4 and Supplemental Table S2. Genetic aberrations included TP53, 1q21, immunoglobulin heavy locus (IGH)/musculoaponeurotic fibrosarcoma, IGH/fibroblast growth factor receptor 3 (FGFR3), and IGH/Cyclin D1 (CCND1) (Supplemental Table S2). Transplanted patient‐derived tumor cells were identified using human‐specific probes/antibodies and enriched in the CD45^dim^/CD38^+^/CD56^+^/CD19^‐^/CD27^+^/CD138^+or−^/cLambda^−^/cKappa^+^ myeloma cell population (Supplemental Figure S5). Serum protein electrophoresis revealed highly expressed free κ‐light chains (Supplemental Figure S6).

Relapsed/refractory multiple myeloma patient‐derived mononuclear cells were then injected subcutaneously into upper forelimbs of NOD scid gamma (NSG) mice. When the tumors reached approximately 40–110 mm^3^, mice were treated with BTZ 0.5 mg/kg (at day 1, 4, 8 and 11) ± CPT 10 mg/kg (daily), after which tumor volumes were measured twice weekly. After 5 weeks, tumor volumes in mice treated with CPT or BTZ alone were significantly reduced compared to untreated controls. However, tumor volumes were significantly diminished following combined CTP/BTZ exposure compared to individual treatment (*p < 0.05 vs. Resistant to bortezomib, **p < 0.01 vs. CPT; Figure 4D). After 5 weeks post‐treatment (Day 36), tumors were excised and imaged (Figure 4E). Western blot analysis performed on tumor specimens revealed that combined CPT/BTZ treatment triggered enhanced PARP cleavage, TRAF3 up‐regulation, and c‐FLIP down‐regulation (Figure 4F), as observed in vitro. Finally, CPT/BTZ treatment induced minimal toxicity and weight loss (*p* > 0.05, Figure 4G), indicating that the CPT/BTZ regimen is active and tolerable in vivo.

## 6 DISCUSSION

Resistant to bortezomib represents a staple for MM treatment at all stages of the disease. Circularly permuted TRAIL a recombinant mutant of human Apo2L/TRAIL, is a novel antitumor candidate for MM and other hematologic malignancies. Resistant to bortezomib promotes apoptosis through the mitochondrial pathway,^26^ while TRAIL mainly acts through the extrinsic apoptotic pathway. Our group and others have shown that concomitant intrinsic and extrinsic apoptotic pathway activation robustly induces apoptosis in malignant cells.^17^ BTZ/TRAIL synergism has been observed in various cell types, including MM.^27^ However, whether such interactions could be extended to CPT, particularly in bortezomib‐resistant MM cells, remains unknown.

The present studies demonstrate that CPT/BTZ synergistically induced apoptosis in diverse MM cell lines for example, U266, H929 and 8226, including BTZ‐resistant PS‐R cells.^28^ However, modestly higher BTZ concentrations were required in the latter (e.g., 10–15 nM vs. 2–5 nM) to achieve synergism, arguing that BTZ resistance is not completely circumvented. Nevertheless, the former concentrations are still low and readily achievable in the plasma.^3^

Death receptors DR4 and DR5 were detected on the surface of both U266 and BTZ‐resistant PS‐R cells after CPT/BTZ treatment. Compared to controls, DR4 endocytosis was markedly increased in cells exposed to both agents, whereas only modest effects on DR5 in both U266 and PS‐R cells, in contrast to findings in solid tumor^29^ and myeloma cells^30^ exposed to BTZ and TRAIL in which DR5 was implicated in cell death. Our findings implicate endocytosis of DR4, but not DR5, in CPT/BTZ‐mediated apoptosis in MM cells.

The ability of TRAIL to activate the extrinsic apoptosis is well described,^31^ and FADD like interleukin‐1‐β converting enzyme inhibitor protein (c‐FLIP), a natural caspase inhibitor protein, regulates extrinsic pathway‐mediated apoptosis. c‐FLIP overexpression inhibits apoptosis mediated by death receptors for example, Fas and TRAIL‐R.^32^, ^33^, ^34^ Increased c‐FLIP expression in PS‐R cells suggests that the alternative apoptotic pathway may be inhibited in BTZ‐resistant cells. Interestingly, CPT/BTZ down‐regulated c‐FLIP, enhanced cleavage/activation of caspase‐8, caspase‐3 and PARP, and up‐regulated FADD. Significantly, CPT/BTZ induced cell death was markedly reduced in dominant‐negative FADD or caspase‐8 cells, indicating that extrinsic cascade activation plays an important functional role in CPT/BTZ‐induced cell death.

The CPT/BTZ regimen activated the intrinsic apoptotic pathway, indicated by caspase‐3 and 9 cleavage, contributing significantly to regimen activity. This pathway has been implicated in bortezomib lethality,^35^ and potentiation by CPT may reflect cooperation between intrinsic and extrinsic apoptosis.^36^ Intrinsic apoptosis is regulated by anti‐apoptotic proteins such as BCL‐2 and BCL‐X_L_)^37^ and the observation that both BCL‐2 and BCL‐X_L_ overexpression protected cells from CPT/BTZ implicates the intrinsic apoptosis in the regimen's activity.

In view of evidence that bortezomib kills malignant cells by interrupting the canonical NF‐κB,^38^ the finding that CPT/BTZ did not inhibit canonical NF‐κB signaling was unanticipated, Instead, combining CPT with BTZ increased phosphorylation and nuclear accumulation of p65, arguing against a role for canonical NF‐κB pathway interruption. Notably, in some malignant hematopoietic cells, BTZ activates this pathway by inducing autophagic degradation of IκBα.^39^

In marked contrast to canonical NF‐κB signaling, the CPT/BTZ regimen diminished non‐canonical pathway activation. TNF receptor associated factors 3 is an inhibitor of non‐classical NF‐κB pathway^40^ and it negatively regulates the NF‐κB inducing kinase (NIK), which represents a key component of the non‐canonical pathway of NF‐κB activation.^41^ Cells treated with CPT and BTZ exhibited pronounced up‐regulation of TRAF3 and down‐regulation of NIK, associated with increased expression of phospho‐p100, reflecting impaired processing of p100 to p52, and diminished expression/nuclear accumulation of p52, hallmarks of non‐canonical NF‐kB activation. Notably, TRAF3 knockdown significantly reduced CPT/BTZ toxicity, collectively arguing that the inhibition of the non‐canonical, but not the canonical NF‐κB pathway, plays a critical functional role in CPT/BTZ activity.

Multiple myeloma cell survival is highly dependent on the bone marrow microenvironment for survival and drug resistance.^42^ Notably, the CPT/BTZ regimen effectively induced cell death in MM cells co‐cultured with human stromal. Non‐canonical pathway activation is implicated in microenvironmental forms of resistance,^17^, ^43^ suggesting that pathway inactivation by CPT/BTZ contributes to stromal cell resistance circumvention.

The CPT/BTZ regimen induced cell death in primary CD138^+^ MM cells, including cells from proteasome inhibitor‐resistant patients. In six of 10 RR MM specimens and two of two newly diagnosed MM samples, primary cells displayed synergistic CPT/BTZ interactions. Unfortunately, the small number of CD138^+^ cells available from these specimens made it impractical to determine whether events responsible for CPT/BTZ synergism in cell lines were operative in primary cells. Future studies may address this question.

The present findings demonstrate that CPT/BTZ co‐administration was well tolerated and effective in a PDX mouse model. Unlike other hematologic malignancies for example, adult acute myeloid leukemia, PDX myeloma models are difficult to generate.^44^ In some cases, genetically humanized mice are required for primary MM cell growth.^44^ Here, primary MM cells were identified that propagated in standard NSG mice. Cell identity was validated using human antibodies and probes consistent with a myeloma origin for example, CD45^dim^/CD38^+^/CD56^+^/CD19^‐^/CD28^+^/CD138^+or−^/cLambda^+^/cKaappa^−^. Importantly, combined CPT/BTZ treatment in vivo induced significantly greater tumor growth inhibition compared with single‐agent administration associated with minimal toxicity. That tumor cells extracted from post‐treatment mice displayed several of the in vitro findings (e.g., PARP cleavage, TRAF3 up‐regulation, and down‐regulation of c‐FLIP) suggests that analogous mechanisms for example, involvement of the extrinsic apoptotic and non‐canonical NF‐κB pathways operate in vivo.

In summary, the present studies indicate that CPT interacts synergistically with BTZ in MM cells, including those resistant to BTZ, through a mechanism involving activation of both the intrinsic and extrinsic apoptotic pathways and inactivation of the non‐canonical NF‐κB cascade. Significantly, similar interactions were observed in primary MM cells, and the CPT/BTZ regimen was active in a MM PDX model with minimal toxicity. Given the introduction of CPT into the clinical arena in MM, these findings provide a theoretical foundation for considering a regimen combining CPT with BTZ in relapsed/refractory MM patients, particularly those resistant to proteasome inhibitors. They also provide mechanistic insights that could guide the rational design of correlative pharmacodynamic assays accompanying future trials.

## AUTHOR CONTRIBUTIONS

Yun Leng, Xiaoyan Hu and Lin Li performed in vitro and in vivo studies, carried out statistical analyses, designed the figures, and wrote the manuscript; Jewel Nkwocha and Kanika Sharma contributed to experimental procedures and checked the original data; Toshihisa Satta collected and analyzed patient samples; Huixing Zhou and Zhiyao Zhang contributed to experimental procedures; Liang Zhou designed the figures and wrote the manuscript; Wenming Chen and Steven Grant conceived and supervised the study and edited the figures and the manuscript.

## CONFLICT OF INTEREST

The authors declare that they have no competing interests.

### PEER REVIEW

The peer review history for this article is available at https://publons.com/publon/10.1002/hon.3045.

## ETHICS STATEMENT

Informed consent was obtained from all patients participating in this study. All animal studies were conducted according to the Ethics Committee of Beijing Chaoyang Hospital and complied with the institutional guidelines for the care and use of animals.

## Supporting information

Supporting Information S1

## Data Availability

All original source data (chiefly Western blot data) linked to the figures in the manuscript are shared on the website OSFHOME. https://osf.io/yu4vs/?view_only=f7f365c2b610497eb3bd9e8799057bc8.
