# Supplementary material for: Mechanisms underlying synergism between circularized tumor necrosis factor‐related apoptosis inducing ligand and bortezomib in bortezomib‐sensitive or ‐resistant myeloma cells
Source: Hematol Oncol. 2022 Jul 14;40(5):999–1008. doi: 10.1002/hon.3045 (PMC10084357; doi:10.1002/hon.3045)

**Supplemental Methods**

**Major reagents**

RPMI-1640 medium (Gibco, USA), Fetal Bovine Serum FBS (Gibco, USA), Dimethyl Sulfoxide (DMSO) (Sigma, USA), Phosphate Buffer Saline (PBS) (Hyclone, USA), 7AAD (Sigma, USA), DioC6 (Invitrogen, USA). Caspase 9 inhibitor Z-LEHD-FMK (S7313, SelleckChem, USA). Immunofluorescence death receptor 4/5 antibody and immunofluorescence binding secondary antibody IgG (HL)-FITC (Santa Cruz, USA), FITC-Annexin V (Thermo Fisher Scientific, USA), Anti-CD-138 immunomagnetic beads (Miltenyi Biotec, USA), CD38 antibody, CD138 antibody and CD45 antibody (Becton Dickinson, USA).

**Analysis of cell death**

Cell death was assessed by staining with 7-AAD as before[19].

**Western blot**

U266 and PSR cells (3～5×106) were treated with different concentrations of CPT (10 ng / ml, 30 ng /ml, 50 ng/ml), TRAIL (50 ng/ml), bortezomib (1.5 nM, 2.0 nM), (PSR cells 10 nM, 15 nM) alone and combination. Whole-cell extracts were prepared from M-PERTM Mammalian Protein Extraction Reagent (Thermo Scientific, Rockford, IL), and quantified using Coomassie Protein Assay Reagent (Pierce ThermoFisher Scientific, Rockford, IL). Equal amounts of protein (20 µg) were separated by SDS-PAGE and electro-transferred onto nitrocellulose membrane. the blots were probed with with antibodies against β-actin (A2066, Sigma-Aldrich, St. Louis, MO), GAPDH (ab9484, Abcam, Cambrige, MA) or α-tubulin (CP06, Calbiochem, La Jolla, CA). The following primary antibodies were purchased from Cell Signaling Technology (Beverly, MA): anti-BCL-2 (4223), anti-BCL-XL (2762S), anti-cleaved Caspase-3 (Asp175, 9661S), anti-PARP (9532S), anti-p100/p52 (4882S), anti-p-p100 (Ser864, 4881), anti-NIK (4944S) and anti-p-p65 (Ser536, 3036S). Anti-c-FLIP (343002) was purchased from Calbiochem (La Jolla, CA). Anti-caspase-8 was purchased from Enzo Life Sciences (ALX-804-242-C100, Farmingdale, NY). Anti-Caspase-3 was purchased from BD (610322, San Jose, CA). Anti-SMAC (cat#05-681) was purchased from Upstate (Lake Placid, NY). The remaining antibodies were purchased from Santa Cruz (Dallas, TX): anti-Caspase-9 (p10, sc-17784), anti-Cytochrome C (A-8, sc-13156), anti-TRAF3 (sc-949), anti-FADD (sc-5559).

**Nuclear extracts**

Nuclear proteins were isolated using a Nuclear Extract kit (Active Motif, Carlsbad, CA) following the suppliers’ instructions. Chemiluminescent DNA-binding ELISA-based assay for activated p65 and p52 DNA binding capacity of NF-κB was determined in U266 cell nuclear extracts using the TransAM® (Active Motif) NF-κB p65 or p52 Chemi Act Assay according to the suppliers’ instructions.

**Detection of TRAIL receptor on the cell surface by immunofluorescence staining**

U266 cells and PS-R cells (5 × 105) were divided into control IgG group, CPT group, BTZ group and combination group, respectively. Subsequently, cells were labeled with anti-mouse IgG (bound to fluorescein isothiocyanate, FITC) and incubated at 4°C in the dark for 1 hour. The nuclei were stained with DAPI. At 16 hours, the distribution of TRAIL receptors on the cell surface and in the cells were detected by fluorescence microscopy.

**Fluorescence in situ hybridization (FISH) analysis**

FISH analysis was performed and analyzed by Kindstar Global® (Beijing, PRC).

**Establishment of a MM patient-derived xenograft (PDX)-bearing mouse model**

Twelve SPF-grade NOD-SCID female mice of 3 to 4 weeks old, weighing about 8.2 ± 1.2 g, were randomly divided into experimental group and control group, with 6 mice in each group. The primary ascites tumor cell suspension was injected subcutaneously into the upper limb side of mice according to 10 × 106 cells/100 ul. The PBS/Matrigel mixture was injected with at the same site in control group. After tumorigenesis (4 weeks), the transplanted tumors were resected and manipulated into a single-cell suspension. Then cells were confirmed to be patient-derived monoclonal plasma cells by flow cytometry (Kindstar Global®), Interphase Fluorescence In Situ Hybridization (FISH) and Serum Protein Electrophoresis (SPE).

**Supplemental Figure Legends**

**Supplemental Figure 1. Combined treatment with CPT and BTZ promotes pronounced DR4 receptor internalization**

(A and B) U266 and PS-R cells were exposed (16 hr) to CPT (30 ng/ml) and BTZ (2 nM in U266 and 10 nM in PS-R) treatment. Cells were stained with DR4 or DR5 with DAPI. Images were obtained with an IX71-Olympus inverted system microscope at 20× magnification.

**Supplemental Figure 2. The CPT/BTZ regimen activates of the extrinsic / intrinsic apoptotic pathway.**

(A) PS-R cells were incubated with CPT ± BTZ for 48 hr. Caspase-8, Caspase-3, PARP, FADD, and c-FLIP were monitored by immunoblotting analysis. CF = cleavage fragment. α-tubulin was assayed to ensure equivalent loading and transfer. (B) PS-R cells were incubated with indicated doses of CPT ± BTZ for 48 hr. Cytochrome C and SMAC were monitored by immunoblotting analysis. (C) PS-R cells were incubated with of CPT ± BTZ for 48 hr. Caspase 9 monitored by immunoblotting analysis.

**Supplemental Figure 3. Combined treatment with CPT and BTZ circumvents microenvironment-driven resistance.**

(A) GFP-labeled PS-R cells co-cultured with or without BM stromal HS-5 cells, and were incubated with LBH ± LCL for 24 hr. Apoptosis of GFP+ cells was analyzed by FCM of 7-AAD staining. (B) Images were obtained with an IX71-Olympus inverted system microscope at 10× magnification.

**Supplemental Figure 4. Interphase fluorescence in situ hybridization (FISH) analysis of bone marrow tissue at diagnosis of RRMM.**

Plasma cells nuclei positive for: (A) 1q21-1q21 probe, (B)17p12.1-TP53 probe, (C-E) 14q32-IGH probe, e, 4p16.3-FGFR3 probe, 11q13-CCND1 probe and 16q23-MAF prob. Fusion-negative for: (C) IGH/FGFR3 probe, (D) IGH/CCDN1 probe and (E) IGH/MAF probe.

**Supplemental Figure 5.** **Representative scatter plots of transplanted tumor cells from mice subjected to flow cytometric immunophenotyping.**

(A) Gated CD45dim cells. (B) CD138/CD38 staining in gated CD45dim cells. (C) CD45/CD38 staining. (D) CD38/CD117 staining in gated CD45dim cells. (E) CD19/CD38 staining in gated CD45dim cells. (F) CD27/CD81 staining in gated CD45dim cells. (G) cLambda/cKappa staining in gated CD45dim cells. (H) CD56/CD19 staining in gated CD45dim cells. (I) CD81/CD38 staining in gated CD45dim cells.

**Supplemental Figure 6. Characterization of heavy chains and light chains in a MM PDX model.** (A) Quantification of human kappa light chain levels at week 4 post-transplant of cells harvested exclusively from the PB in MM PDX mouse plasma. (B) Serum protein electrophoresis (SPE) was performed to monitor levels of IgG, IgA, IgM, Κ light chains, and λ light chains,

**Supplemental Table S1. Clinical information for primary MM samples.**

**Supplemental Table S2. Genetic information for primary MM sample (PDX).**

**Supplemental Table S1. Clinical information of primary MM samples**

**Abbreviation**:

**PC**=plasma cells; **RR**=relapsed or refractory; **ND**=new diagnosis;

**FISH**=Fluorescence In Situ Hybridization; **CI**=combination index.

**PBSCT**: peripheral blood stem cell transplantation; **PAD**: bortezomib+doxorubicin+dexamethasone;

**CTD**: cyclophosphamide+thalidomide+dexamethasone; **PD**: pomalidomide+dexamethasone;

**VAD**: vincristine+doxorubicin+dexamethasone; **PDD**: bortezomib+liposome doxorubicin+dexamethasone;

**RD**: lenalidomide+dexamethasone; **VRD**: bortezomib+lenalidomide+dexamethasone;

**RCD**: lenalidomide+cyclophosphamide+dexamethasone; **PCD**: bortezomib+cyclophosphamide+dexamethasone;

**TD**: thalidomide+dexamethasone; **PTD**: bortezomib+thalidomide+dexamethasone;

**PCDT** : bortezomib+cyclophosphamide+thalidomide+dexamethasone; **KAD**: carfilzomib+doxorubicin+dexmathasone;

**M2**: melphalan+carmustine+cyclophosphamide+vincristine+prednisone; **MP**: melphalan+prednisone;

**VRCD**: bortezomib+lenalidomide+cyclophosphamide+dexamethasone;

**VCED**: bortezomib+cyclophosphamide+etoposide+dexamethasone;

**VDT-PACE**: bortezomib+dexamethasone+thalidomide+cisplatin+doxorubicin+cyclophosphamide+etoposide.

**Supplemental Table S2. Genetic information of primary MM sample (PDX)**

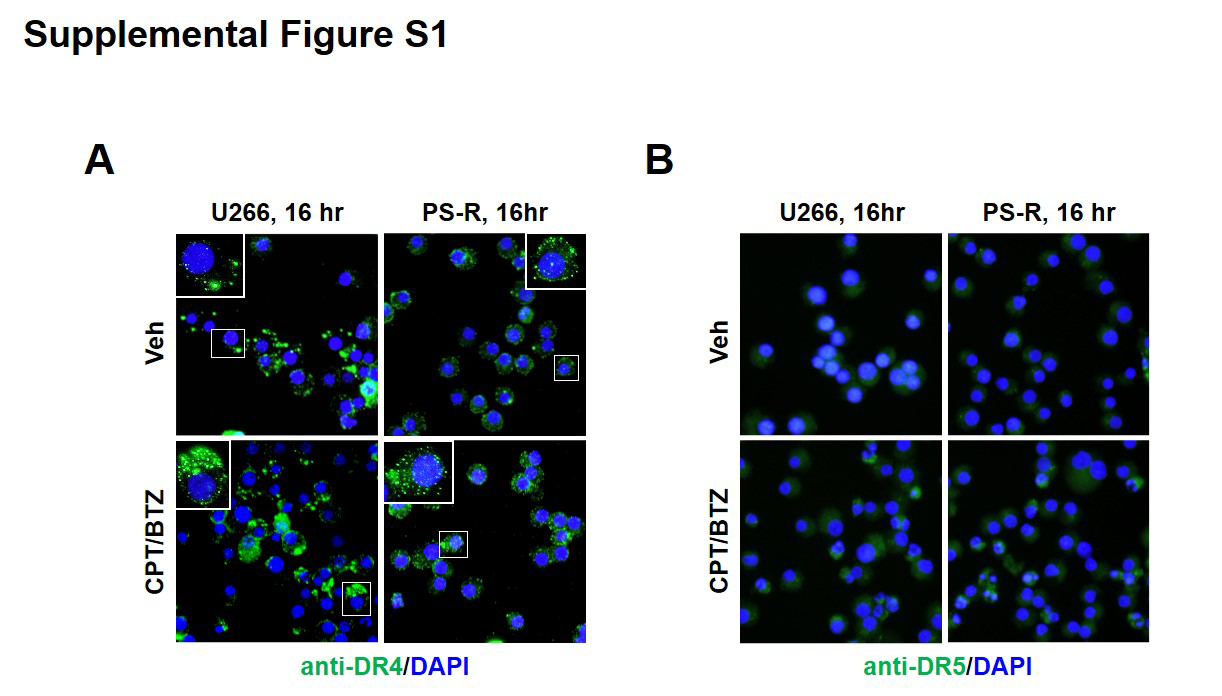


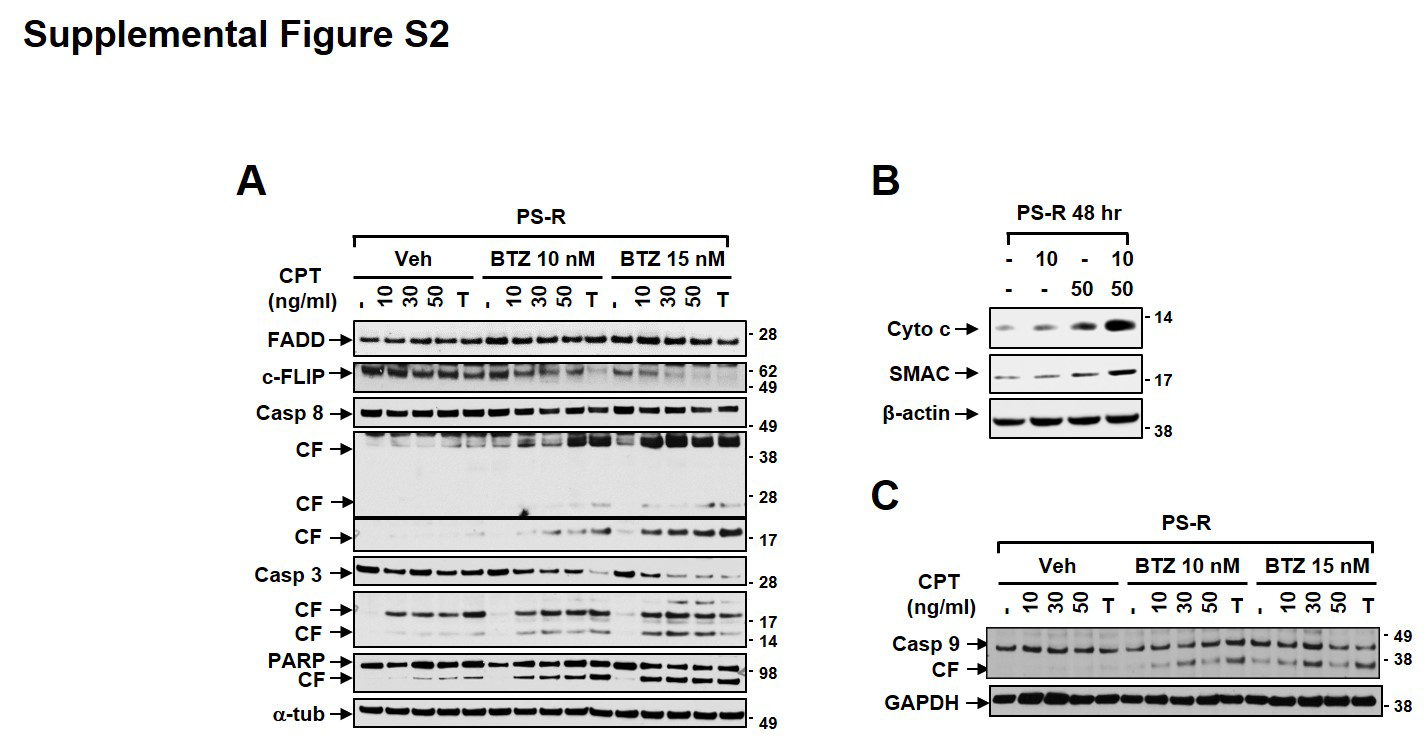


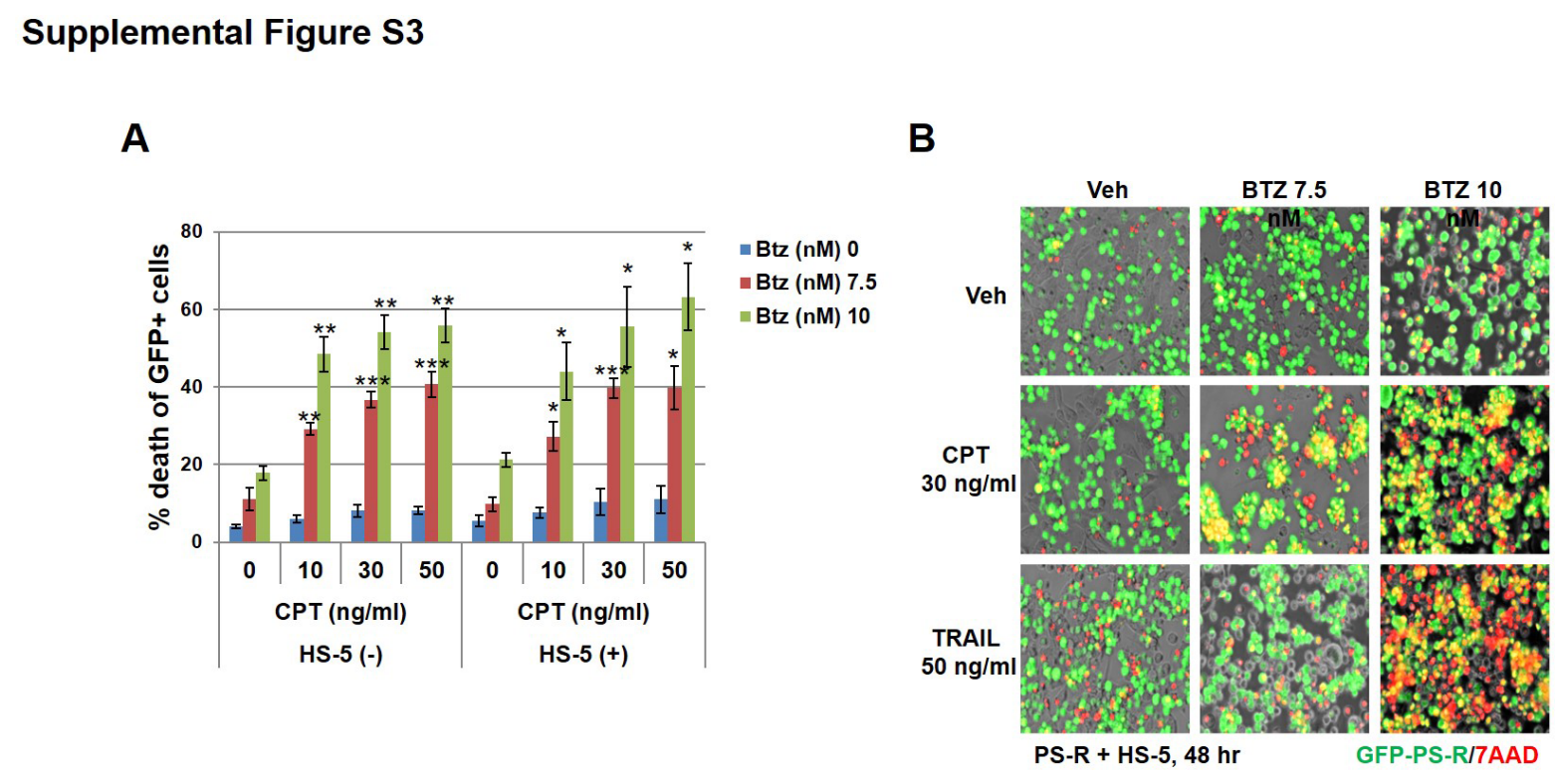


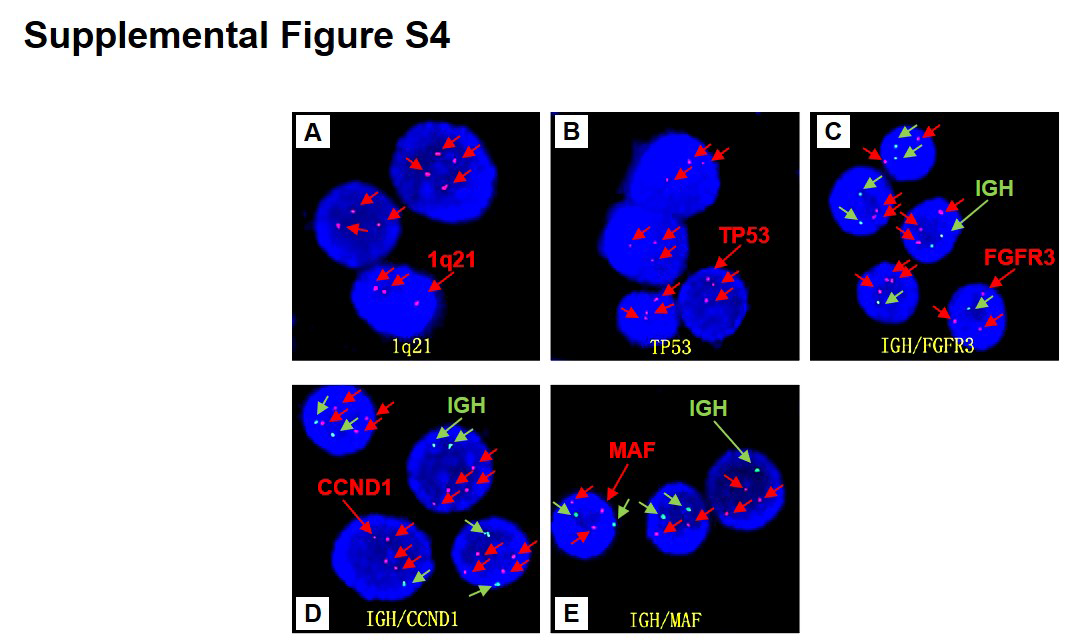

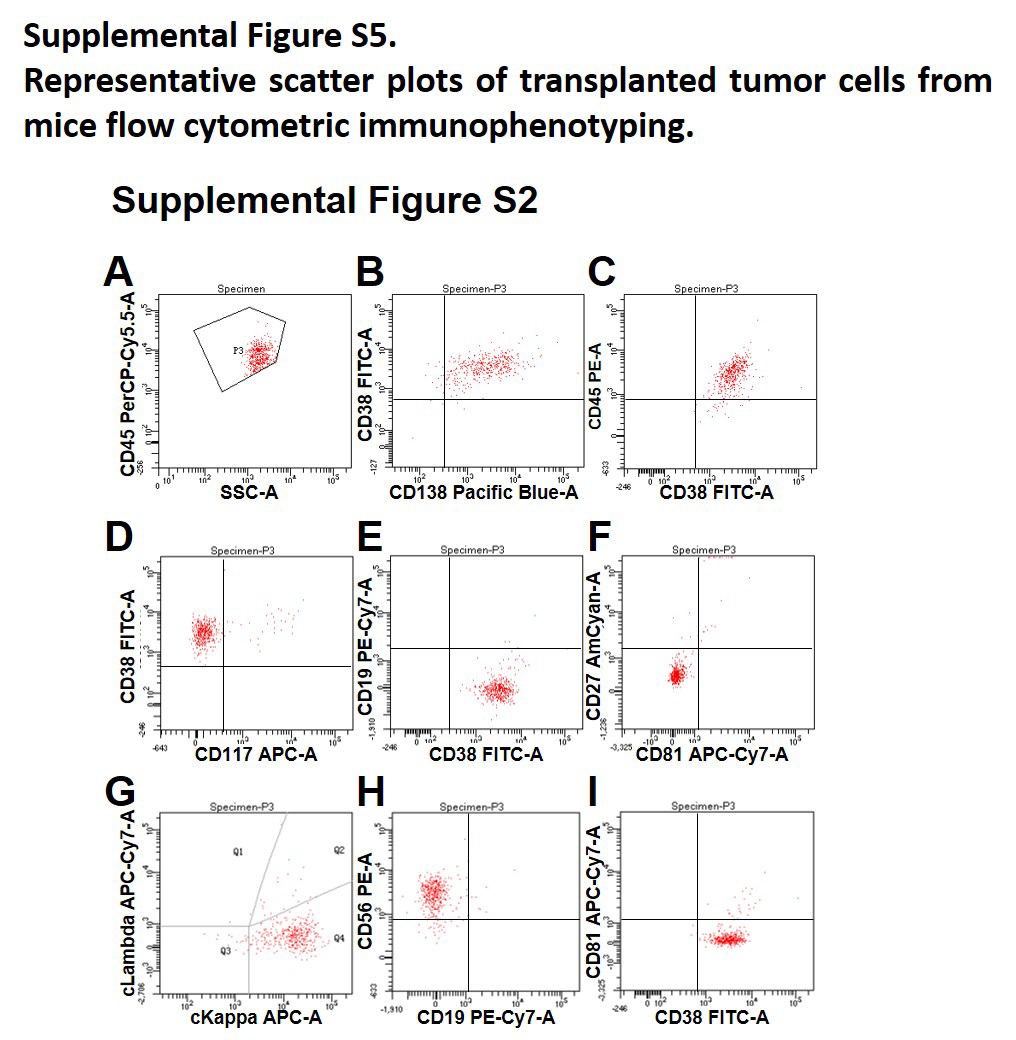


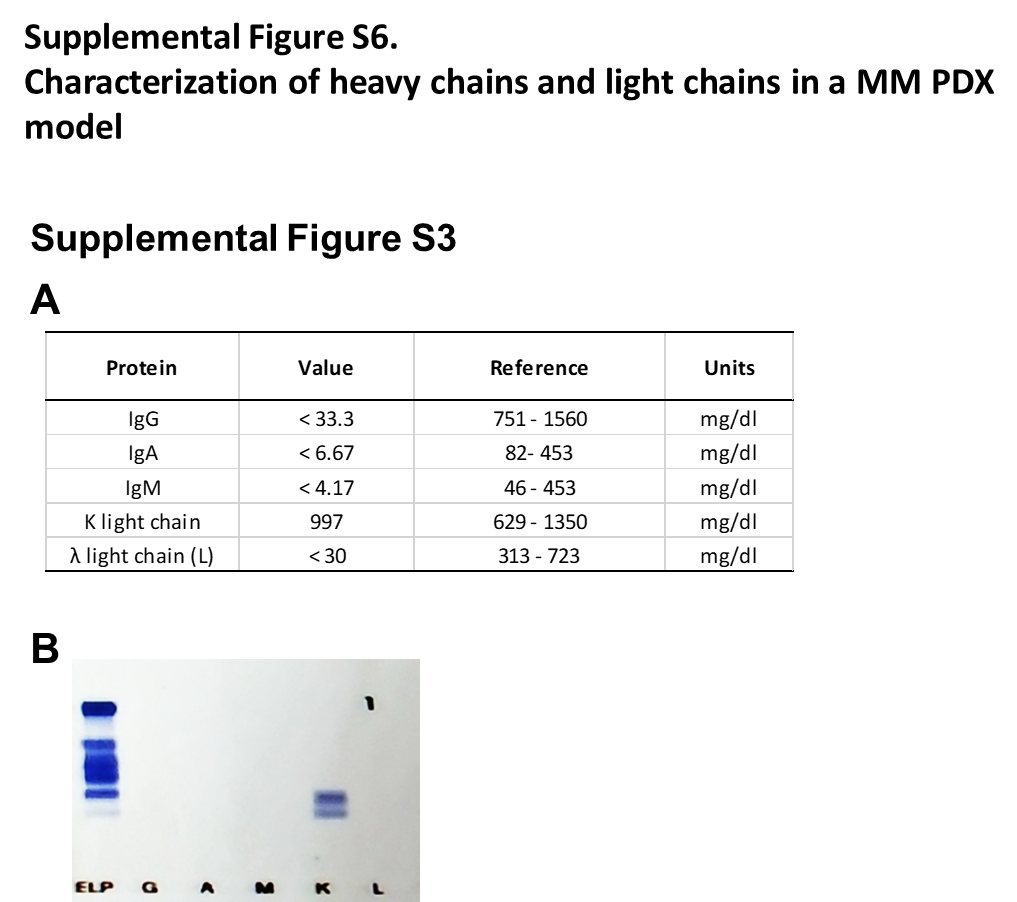

Supplement: Supplementary file 1 — Supporting Information S1 [file HON-40-999-s001.docx]
